# Unveiling motives for dentistry studies: psychometric validation of a comprehensive questionnaire among aspiring dental students

**DOI:** 10.1038/s41405-024-00208-5

**Published:** 2024-03-28

**Authors:** Jorge Moncayo-Rizzo, Geovanny Alvarado-Villa, Iván Cherrez-Ojeda, Juan Carlos Gallardo, Eleonor Velez Leon, Susana Patricia Gonzalez Eras

**Affiliations:** 1 Universidad Espíritu Santo, 0901952 Samborondón, Ecuador; 2 Universidad Católica de Santiago de Guayaquil, 090615 Guayaquil, Ecuador; 3 Universidad Católica de Cuenca, 010102 Cuenca, Ecuador; 4 Universidad Nacional de Loja, 110111 Loja, Ecuador

**Keywords:** Extended skills training in dentistry, Dental clinical teaching

## Abstract

**Objective:**

The aim of this study is to develop and validate a questionnaire for dental students in Ecuador to assess their reasons to study dentistry.

**Materials and methods:**

A 25-item questionnaire was developed by the authors based on similar studies and a qualitative study. The questionnaire had five theoretical factors: economic, professional, vocational, social and academic reasons for study. In the first two samples, exploratory factor analysis was performed to identify the structure, and the models obtained were compared with confirmatory factor analysis in the third sample.

**Results:**

Three samples were used, sample A with 201 participants, sample B with 623 participants, and sample C with 596 participants. Two-thirds of the participants were female and almost one-third were from coast region. The EFA applied in sample A resulted in a five-factor model with 18 items; in contrast, a three-factor model was obtained from sample B. According to the CFA in sample C, the best model was explained by three factors: labor, vocational and academic reasons. Considering two items to cross-load in labor and vocational factors, which are theoretically justified.

**Conclusion:**

This study presents a 12-item questionnaire that assesses labor, vocational and academic reasons for studying dentistry in an Ecuadorian population.

## Introduction

In recent years, there has been a global surge in the demand for healthcare professionals, possibly driven by their esteemed status as in-demand vocations [1–3]. This trend extends to the field of dentistry where studies have reported an unprecedented increase in dental school enrollment demands and a notable shift in gender ratios within the workforce [4, 5]. Therefore, a thorough exploration of the motivations behind the pursuit of dentistry becomes imperative, not only to facilitate the design of effective recruitment strategies and dental curricula, but also to provide prospective students with a comprehensive understanding of the profession before committing to it.

Moreover, there are some differences in the field of dentistry at a regional level. Currently, a new focus is being introduced within the dental curriculum, known as Special Needs Dentistry. A study conducted by Scepanovic et al. found that despite South America having the highest number of dental schools, none of them incorporate this new focus, highlighting the disparity in the educational advancement of dentistry [6].

In several studies, factors that influence the decision to pursue dentistry as a career were assessed. Significant research emerges from qualitative studies, aiding in the identification of the distinct constructs that shape the decision to pursue dentistry as a career. Gallagher et al.’s research, for instance, emphasizes the importance of professional and financial incentives in dentistry, as well as student life [7]. Gallagher et al.’s research, for instance, underscores the significance of professional and financial incentives in dentistry, as well as the stresses associated with student life [4, 8]. These factors are assessed by different quantitative studies as vocational factors and personal factors, or third-person influences [3, 9–11].

While motivations for selecting dentistry as a career have been extensively studied on a global scale [2, 3, 12–16]; there is a notable scarcity of research on this subject within Latin American countries. According to the literature, there are only four studies about this topic from Brazil and one from Peru [9, 17–20]. Despite the commonality of this research focus, no standardized and validated questionnaire currently exists to comprehensively assess the motivations of dental students in their choice of career. Existing questionnaires have either been derived from similar instruments or developed through expert consensus. A limited number of studies have employed validation methods beyond content and face validation [9, 10, 21, 22]. Therefore, there are no studies assessing construct validation of these questionnaires in Latin America, which is one of the steps when developing a questionnaire according to the best practices for developing and validating scales for health [23].

In accordance with the framework outlined by Kishore et al. [24] and Boateng et al. [23], the process of questionnaire development encompasses several crucial stages, including construct validity, convergent validity, factor analysis, internal consistency, and descriptive analysis, among others [24]. The same processes must be considered when translating a questionnaire to another language, as it is necessary to ensure that the concepts have not changed [24]. Thus, the aim of this research is to validate a questionnaire constructed to evaluate the factors influencing the decision to pursue a career in dentistry.

## Materials and methods

### Questionnaire design

The questionnaire was developed by the research team. The questions were selected from similar studies [9, 10, 12, 21], and qualitative studies [7]. Additionally, questions were incorporated to align with the Ecuadorian academic culture. As a result, a 25-item questionnaire was developed, encompassing five theoretical factors that were supported by the qualitative studies. These factors encompassed economic (Q1–Q4), professional (Q5–Q9), vocational (Q10–Q14), social (Q15–Q20), and academic motivations (Q21–25) for choosing dentistry. Each item was rated from 1 to 5 on a Likert scale (from totally disagree to totally agree). This questionnaire was evaluated in three samples to assess its validity.

According to Gallagher’s study [7], several factors were identified after a qualitative analysis. These factors were ’professional status,’ ‘financial benefits,’ ‘job security, flexibility and independence,’ ‘good quality of life,’ ‘personal experiences’ and ‘alternative career considered’. In determining the questionnaire items, the authors meticulously analyzed and selected questions from similar questionnaires [9, 10, 21], which may fit on each factor. Moreover, as mention by Gallagher, the status of a professional in a social and economic order involve job security, with a regular income, and independence [7, 25]. According to this, we mixed the ‘job security, flexibility and independence’ factor with ‘professional status,’ creating a single factor named ‘professional reasons.’ [7, 25, 26]. The ‘good quality of life’ factor was merged with ‘financial benefits’ to create a new factor named ‘economic reasons.’ [7, 25]. ‘Personal experiences’ factor was named ‘social reasons’ and it considered the influences of family and friends and the contact with the odontology environment [7, 25]. Finally, ‘alternative career considered’ was named ‘Academic reasons’ which assess the fact that some students did not reach enough points to choose other careers (generally, medicine) [7, 25]. Also, here we added a question about a test taken by the government on the last high school year which contributes in 60% to the score which is postulated to access superior education [27].

The ethical approval for this study was granted by the Hospital Clinic Kennedy Ethics Committee, with the reference number HCK-CEISH-2022-002.

### Sample

The present study was performed with three samples. Sample A consisted of 201 participants, while samples B and C included 623 and 596 students, respectively. The response rate for samples B and C was 81%. Participants were sourced through faculty members from the dentistry departments of several private universities in Ecuador. The samples were chosen using a nonrandom convenience method. Sample A was collected from April 25 to May 27; sample B was collected from May 30 to June 4; finally, Sample C was collected from June 27 to July 31, 2022. This sampling method was employed due to the ease and rapidity of data collection. Moreover, given the study’s objectives, the representativeness of subjects from the country takes a back seat to prioritizing the accurate formation of the construct. For questionnaire distribution, it was disseminated via email to colleagues nationwide holding teaching positions at private universities with an active dentistry program. In this manner, the survey was made available to students. This recruitment procedure was consistently applied across all three samples.

### Data analysis

Data was processed using the SPSS software for Windows, version 25. Qualitative variables are represented as percentages, while quantitative data are expressed in terms of mean and standard deviation. Both exploratory and confirmatory factor analyses were conducted on the three samples to ascertain the number of factors and the distribution of items within them. Pertaining to the EFA, Bartlett’s test of sphericity and the Kaiser–Meyer–Olkin measure were utilized to determine the sample’s suitability for factor analysis. The Bartlett’s sphericity test assesses the factorability of the correlation matrix, indicating that it was generated by random data. The Kaise–Mayer–Olkin assesses the extent to which correlations are a function of the variance shared across all variables rather than the variance shared by particular pairs of variables. The extraction method employed was maximum likelihood, with an oblique (Oblimin) rotation subsequently applied, assuming a relationship between the factors. Furthermore, inter-item correlations from the derived model were evaluated using Spearman’s rank correlation test due to non-normal distribution of the data [28]. In the context of the CFA, both goodness-of-fit and parsimony indices were computed to discern the most fitting model. These parameters included: CMIN/dF (≤3), CFI (>0.9), GFI (>0.9), RMSEA (<0.06), SRMR (<0.08), AIC and BIC (the lowest score) [29].

The factor analysis was conducted in a sequential manner. First, the distribution of the 25-item questionnaire was explored in the initial sample (sample A, with 201 participants). The questionnaire, which was refined based on the analysis of sample A, was then given to a larger group, sample B, consisting of 623 participants, to evaluate item distribution. After this, a confirmatory factor analysis was carried out with sample C, which had 596 participants.

## Results

### Participants characteristics

Regarding the characteristics of the participants, females were the predominant group in the three samples. The mean age of the participants in sample A was 20.75 years old (SD: 2.57), ranging from age 17 to 32. In sample B, the mean age was 21.32 (SD: 2.79), ranging from 17 to 36 years old. Finally, in sample C, the mean age was 21.81 (SD: 3.2), with an age range of 17 to 42. Almost two thirds of the participants study in the highland region (70.42%). Finally, most of the participants were in their first year of study (29.20%). Table 1 presents the characteristics of the participants from each sample.

**Table 1:** Characteristics of the participants.

		Sample A	Sample B	Sample C	Total
		*n* = 201 (%)	*n* = 623(%)	*n* = 596(%)	*N* = 1420 (%)
Sex	Male	61 (30.3)	202 (32.4)	197 (33.1)	460 (32.4)
	Female	140 (69.7)	421 (67.6)	399 (66.9)	960 (67.6)
Region	Coast	105 (52.2)	179 (28.7)	157 (26.3)	441 (31.1)
	Highlands	93 (46.3)	410 (65.8)	403 (67.6)	906 (63.8)
	Other	3 (1.5)	34 (5.5)	36 (6)	73 (5.1)
Year on the dentistry faculty	1st year (1st and 2nd semester)	89 (44.3)	179 (28.7)	146 (24.5)	414 (29.2)
	2nd year (3rd y 4th semester)	40 (19.9)	128 (20.5)	115 (19.3)	283 (19.9)
	3rd year (5th y 6th semester)	28 (13.9)	138 (22.2)	121 (20.3)	287 (20.2)
	4th year (7th y 8th semester)	33 (16.4)	114 (18.3)	130 (21.8)	277 (19.5)
	5th year (9th y 10th semester)	11 (5.5)	64 (10.3)	84 (14.1)	159 (11.2)

### Exploratory factor analysis in sample A

The whole questionnaire (25 items) was applied to sample A, and EFA was performed to determine the factorial structure of the questionnaire. The Kaiser–Mayer–Olkin (KMO) test was 0.830 and Bartlett’s sphericity test was x^2^: 1301.88; *p* < 0.001, indicating that the sample was suitable for the EFA. The extraction method was maximum likelihood (ML) with Oblimin rotation. The analysis showed five factors that explained 64.56% of the variance.

The first factor (vocational reasons) was formed by items 10–14; the second factor (economic reasons) included items 1–4; the third factor (academic reasons) included items 21, 23, 24; fourth factor (professional reasons) was formed by items 5, 7 and 8; finally, fifth factor (social reasons) included items 15, 18, 20. All of these items had a high loading (>0.4) for each factor, with a minimum of 0.508 and a maximum of 0.834. The explained variance for each factor is presented in Table 2. As a result, the questionnaire was reduced to a five-factor model (Model A) with 18 items.

**Table 2:** Structure matrix of the questionnaire applied in Sample A.

	Factor					Explained variance (%)	Cronbach Alpha
	1	2	3	4	5		
Q12 - Dentistry allows me to work with my hands	0.834	–	–	–	–	29.02	0.838
Q10 - It allows me to help people improve their health.	0.786	–	–	–	–		
Q11 - Improving people’s appearance.	0.762	–	–	–	–		
Q14 - I enjoy working with people and taking care of them.	0.738	–	–	–	–		
Q13 - Dentistry aligns with my artistic talent.	0.677	–	–	–	–		
Q3 - It will allow me to access better compensation.	–	0.822	–	–	–	11.54	0.825
Q2 - It will allow me to have a good standard of living.	–	0.748	–	–	–		
Q4 - It allows me to have greater job stability.	–	0.748	–	–	–		
Q1 - Ease of finding work.	–	0.674	–	–	–		
Q21 - My school grades qualified me to choose dentistry as a career.	–	–	0.628	–	–	10.68	0.571
Q23 - The vocational test I took suggested studying dentistry.	–	–	0.575	–	–		
Q24 - My score in the high school graduation exam qualified me for the career.	–	–	0.559	–	–		
Q5 - It allows me to have my own business.	–	–	–	0.878	–	7.15	0.718
Q7 - I can be my own boss.	–	–	–	0.687	–		
Q8 - I can have a more regular schedule compared to other health professions.	–	–	–	0.518	–		
Q20 - I have been involved with dentistry work.	–	–	–	–	0.641	6.18	0.568
Q15 - One of my relatives or friends is a dentist and encouraged me to study dentistry.	–	–	–	–	0.628		
Q18 - It allows me to work in a team.	–	–	–	–	0.508		
Q6^a^ - I can practice dentistry after graduating without needing to be a specialist.	–	–	–	–	–	–	–
Q9^a^ - Dentists usually do not face life–threatening situations with patients.	–	–	–	–	–		
Q13^a^ - Dentistry aligns with my artistic talent.	–	–	–	–	–		
Q16^a^ - I studied dentistry due to my family’s desire.	–	–	–	–	–		
Q17^a^ - Dentistry is a prestigious profession.	–	–	–	–	–		
Q19^a^ - I had a preference for another career and decided on dentistry.	–	–	–	–	–		
Q22^a^ - I received a scholarship to study dentistry.	–	–	–	–	–		
Q25^a^ - It allows me to achieve a higher academic level.	–	–	–	–	–		

Model A.

^a^Items eliminated due to low communality.

The factors were positively correlated as shown by the Oblimin rotation. Additionally, all the items of the factors are intercorrelated (0.1 < r < 0.9) (Supplementary Table S1). In addition, the reliability, measured with Cronbach’s alpha, was good for vocational, economic and professional factors. On the other hand, academic and social factors have a weak Cronbach’s alpha (See Table 2).

### Exploratory factor analysis in sample B

The 18-item questionnaire was applied to samples B and C. We performed a new EFA on sample B and evaluated the behavior of the items and the factors in this larger sample. The KMO test was 0.850, and Bartlett’s sphericity test was x^2^: 2050.86; *p* < 0.001, indicating that the sample was appropriate for the EFA. Extraction with the ML method and the Oblimin rotation showed a two-factor model that explained 57.92% of the variance.

**Fig. 1:**
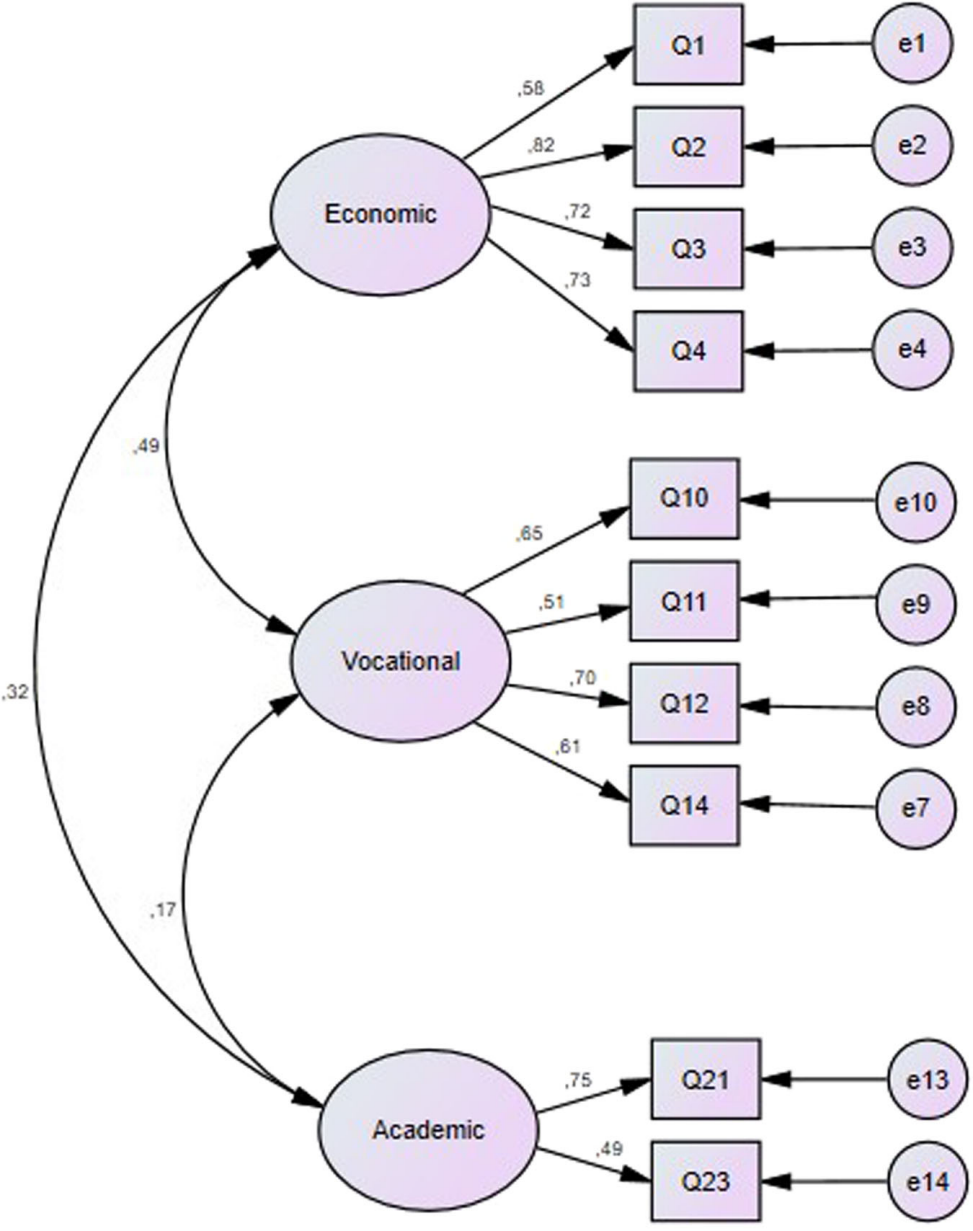
Model B – 10 items distributed in a three-factor model which assess economic, vocational and academic reasons for studying dentistry without cross-loads items.

Item 8 was removed from the analysis due to its low communality (0.197), indicating that the item shares little variance with the other variables. Consequently, it was identified for elimination. Meanwhile, items 5 and 7 have cross-loading on factors one and two with high indexes (from 0.471 to 0.565). Also, due to academic culture influence, the authors allowed academic reasons factor to be formed by two items. As a result, Model B is composed by three factors without cross-loadings items. The first factor (economic reasons) included items 1–4; the second factor (vocational reasons) included items 10–12 and 14; finally, the third factor (academic reasons) was formed by items 21 and 23. So, this model was built up of 10 items (see Fig. 1).

**Fig. 2:**
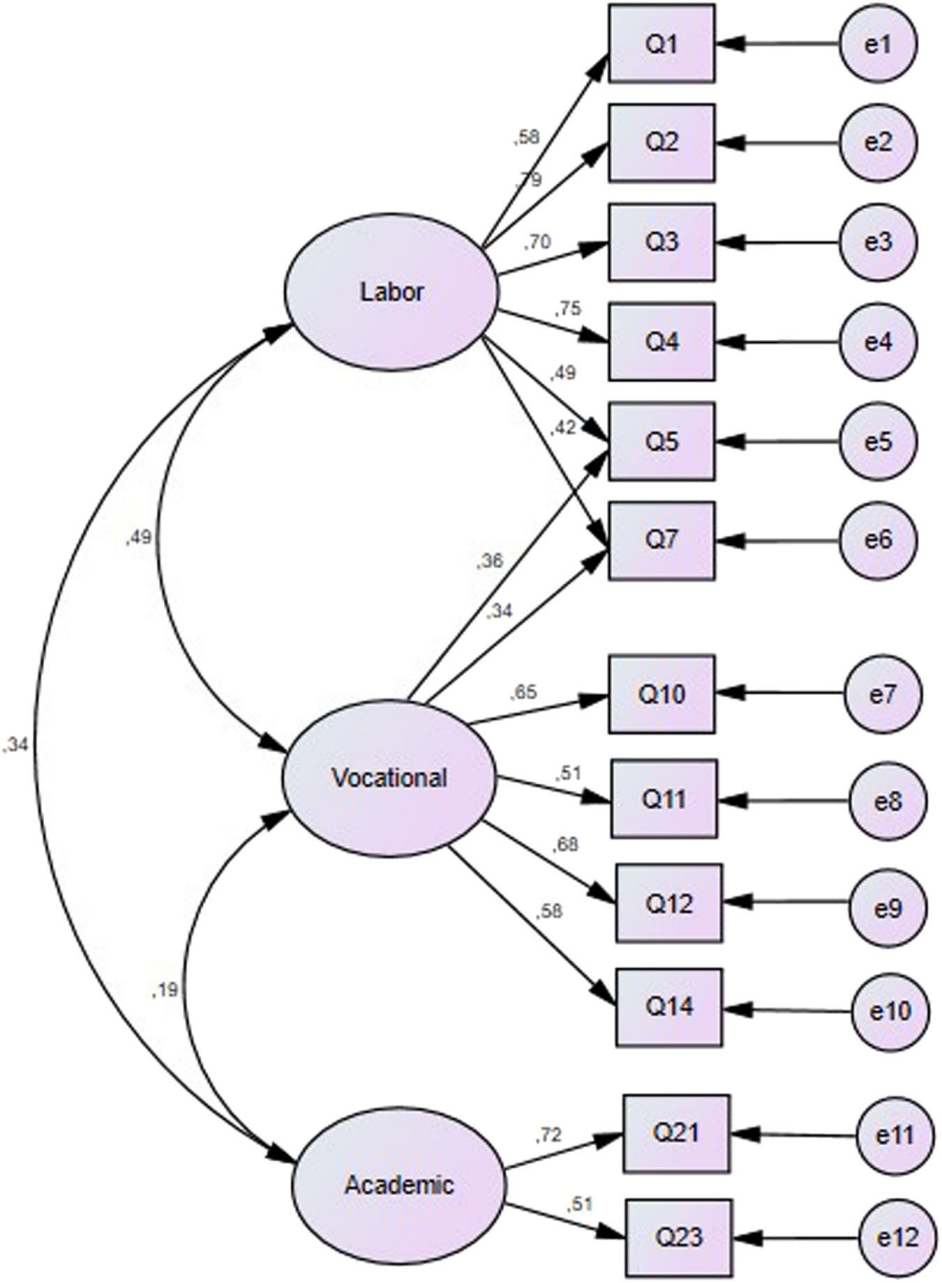
Model C – 12 items distributed in a three-factor model which assess labor, vocational and academic reasons for studying dentistry, allowing two cross-loads items.

However, due to theoretical reasons the authors decided to analyze the influence of cross-loadings [2–4, 30]. The model with cross-loadings (Model C) is composed by three factors. The first factor (labor reasons) included items 1–5, 7; the second factor (vocational reasons) included items 5, 7, 10–12 and 14; finally, the third factor (academic reasons) was formed by items 21 and 23. So, Model C was built up of 12 items (see Fig. 2).

The factors are positively correlated in both models, as shown by the Oblimin rotation. Additionally, all the items of the factors are intercorrelated (0.1 < r < 0.9) (Supplementary Tables S2 and S3). Finally, the reliability of the two scales was acceptable according to Cronbach’s alpha (see Table 3).

**Table 3:** Structure matrix of the questionnaire applied in Sample B.

	Factor			Model B		Model C			
	1	2	3	Variance (%)	Cronbach Alpha	Variance (%)		Cronbach Alpha	
Q4 - Me permite tener una mayor estabilidad laboral *It allows me to have greater job stability*	0.781	–	–	32.98	0.807	33.4	–	0.817	–
Q2 - Me permitirá acceder a una buena condición de vida *It will allow me to access a good living condition*	0.768	–	–						
Q3 - Me permitirá acceder a una mejor remuneración *It will allow me to access a better remuneration*	0.723	–	–						
Q1 - Facilidad para encontrar trabajo *Easy of finding work*	0.611	–	–						
Q5^a^ - Me permite tener mi propio negocio *It allows me to have my own business*	0.565	0.495	–	–	–		13.93		0.769
Q7^a^ - Puedo ser mi propio jefe *I can be my own boss*	0.471	0.508	–						
Q10 - Me permite ayudar a las personas a mejorar su salud *It allows me to help people improve their health*	–	0.737	–	16.62	0.734	–		–	
Q12 - La odontología me permite trabajar con mis manos *Dentistry allows me to work with my hands*	–	0.659	–						
Q11 - Mejorar la apariencia de las personas *To improve people’s appearance*	–	0.609	–						
Q14 - Me gusta trabajar con la gente y cuidar de ellos *I like to work with people and take care of them*	–	0.566	–						
Q23 - El test vocacional que me realizaron me sugería estudiar odontología *The vocational test suggested I study dentistry*	–	–	0.597	12.48	0.523	10.59		0.523	
Q21 - Mis notas del colegio me han calificado para elegir odontología como carrera *My grades from school have qualified me to choose dentistry as a career*	–	–	0.594						

^a^Cross-loading items.

### Confirmatory factor analysis in sample C

**Fig. 3:**
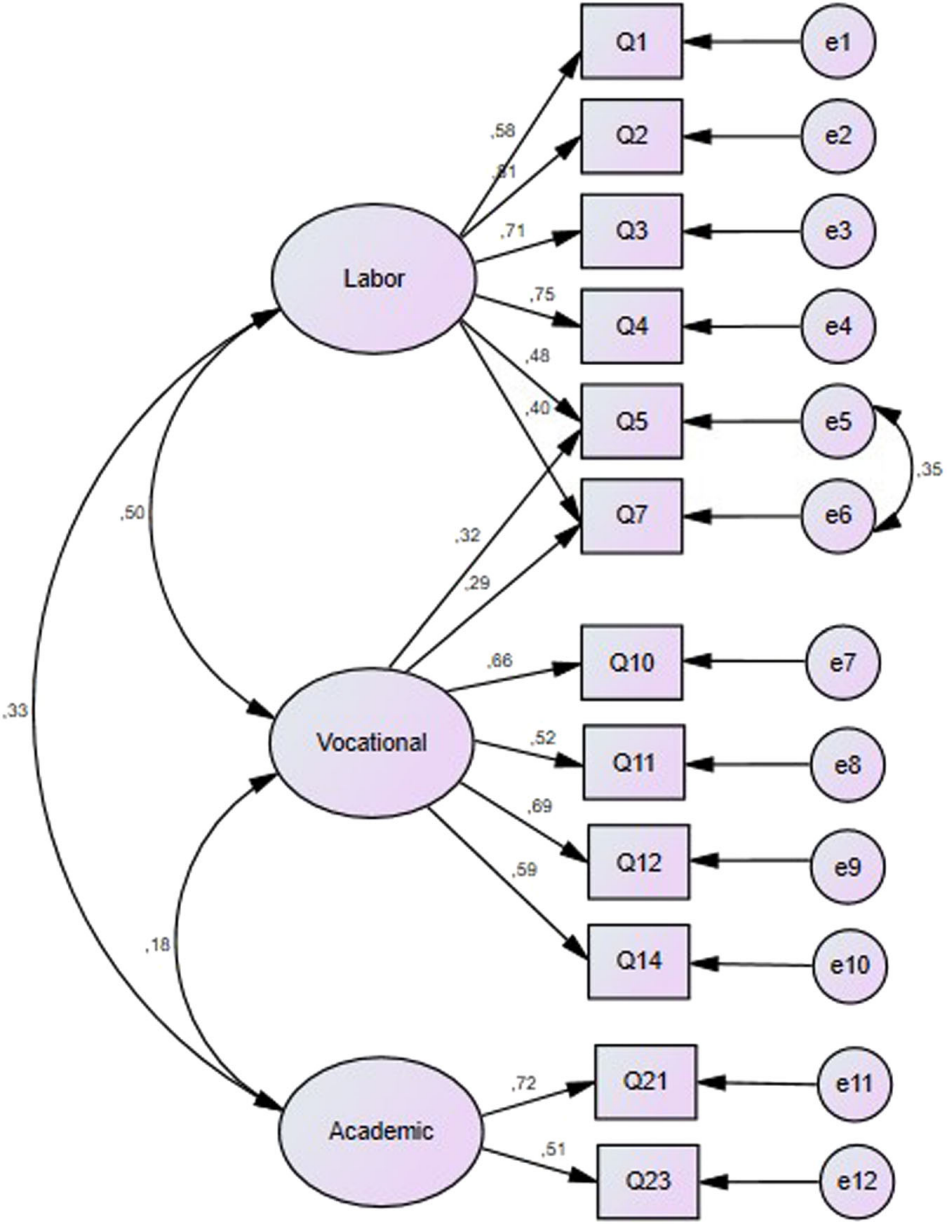
Model D – a three-factor model which allows error correlation from items Q5 and Q7.

The structure proposed by the results of EFA on samples A and B were confirmed with a CFA. This analysis was performed on sample C. Four models were compared: a five-factor model (Model A from sample A), a three-factor model without items exhibiting cross-loading (Model B from sample B), and three-factor model with items exhibiting cross-loading (Model C from sample B), and a three-factor model with cross-loadings and error correlations, for items 5 and 7_,_ (Model D) (see Fig. 3). Table 4 shows the results of the CFA.

**Table 4:** Confirmatory factor analysis.

Model	CMIN/dF	CFI	GFI	RMSEA (CI)	SRMR	AIC	BIC
Model A^a^	3.165	0.904	0.931	0.060 (0.054–0.067)	0.0763	487.671	689.622
Model B^b^	2.500	0.966	0.973	0.050 (0.037–0.064)	0.0408	125.994	226.970
Model C^c^	3.344	0.944	0.956	0.063 (0.052–0.074)	0.0411	221.838	498.439
Model D^d^	2.126	0.974	0.972	0.044 (0.032–0.055)	0.0370	162.066	293.773

^a^Five-factor model.

^b^Three-factor model without cross-loads.

^c^Three-factor model with cross-loads.

^d^Three-factor model with cross-loads and item error correlation.

## Discussion and conclusion

Study motivation of dental students have been a research topic for a long period. For example, Vigild and Schwartz analyzed the behavior of the motivation in Danish dental students from 1972 to 1994 in three cohorts every 10 years [22]. During that period, altruistic reasons strongly influenced dental students’ motivation. Currently, researchers commonly highlight the impact of economic benefits, job prospects, and recommendations from family and friends on students’ motivation to pursue dentistry [12, 14, 31–34]. Furthermore, Niven et al. suggested a dentistry career decision-making pathway based on a qualitative study in secondary schools’ students [35]. This pathway suggests four phases in the decision-making process, which begins with interest in science, continues with social and family influences, personal experiences and finally job and work conditions [35]. Moreover, it has already been proposed that career choice may be influenced by parents’ expectations and desires [36, 37].

Despite the widespread interest in understanding motivations for studying dentistry, the availability of validated questionnaires that comprehensively assess the underlying constructs of students’ motivations remains limited. According to the literature, many of the questionnaires used for these studies are based on questions from other studies that were approved by experts in the field [2, 9, 11, 14, 32, 33], or are based on previous qualitative research [3–5, 7, 8, 10, 11]. Few articles have explored the construct of the questionnaires [9, 10, 21, 22]. The present study analyzes the validity of a questionnaire from a theoretical frame work and a complete factor analysis, including exploratory and confirmatory factor analysis.

The analysis yielded four models. The initial five-factor model aligned with the theoretical framework [4, 5, 7, 10]. Yet, when applied to a larger sample, two changes emerged. Firstly, the ‘social reasons’ factor was eliminated due to its insufficient variance explanation (refer to Table 2) and its items’ low communalities. Secondly, items from ‘professional reasons’ cross-loaded with both economic and vocational reasons, a change supported by the conceptual framework. Many authors have identified a singular ‘job characteristic’ factor encompassing economic benefits and professional advantages [14, 21, 31, 32], leading us to label this factor as ‘labor reasons’.

Furthermore, we permitted cross-loadings of ‘professional reasons’ items into the ‘vocational reason’ factor. Given the evolving nature of vocational reasons, which now often encompass professional and economic aspects as discussed by Chate [30], this interaction was deemed appropriate. Literature supports the significant influence of labor reasons among students [2–4, 12]. For instance, studies have highlighted the appeal of the dental profession’s positive image [2, 4], and the work-life balance it offers [3]. These reasons can be categorized as professional, while motivations like aiding the underprivileged align with vocational reasons [3].

Our findings resonate with Folayan et al., where economic and vocational factors were pivotal [21]. However, we diverged in our consideration of parents’ recommendation due to its reported influence on choosing dentistry [4, 11, 12, 14, 33]. The ‘social reasons’ factor, which was ultimately excluded, initially encompassed friends and parents’ recommendations and exposure to the dental environment.

Comparing our results with Folayan et al., we noted differences in the profession-related factor [21]. We categorized items related to job security and income under economic reasons, while the social status item was excluded due to its low communality (Q17). Our exploratory factor analysis indicated a trend of professional reasons items loading into economic reasons, resulting in a unified ‘labor reasons’ factor.

Bernabé et al.’s study identified four factors with 16 items [9]. However, the sample size was insufficient for a reliable EFA interpretation [38], necessitating a CFA for model confirmation [39, 40]. Without access to their factor loadings, a direct comparison was challenging.

Contrastingly, three studies proposed an eight-factor model [10, 21, 41]. Our findings, along with those from Rashid et al. [10], Vigild et al. [22], and Scarbecz et al. [41], consistently highlighted economic and vocational reasons. Scarbecz et al.’s CFA presented a four-factor model closely mirroring our findings [41].

Despite its weak Cronbach’s alpha, we retained the ‘Academic reasons’ factor, given reports of students opting for dentistry as a secondary choice [4, 5, 32, 33]. The CFA led to the exclusion of the five-factor model, leaving three models for comparison. Model A1 emerged as the most fitting, especially since Q5 and Q7 shared a theoretical foundation.

This study has limitations, including its focus on private universities in Ecuador, making the results non-generalizable to all dental students or other Latin American countries. Additionally, factors such as gender, region, and academic year were not analyzed for response homogeneity within or across the samples. Despite identifying the motivations for studying dentistry, the intersection of professional and economic interests may result in changes in students’ motivations. Therefore, motives for studying dentistry should be explored over time. However, the study’s strength lies in its large and adequate sample size, as well as diverse representation from various university. Moreover, the step-by-step validation process adheres to the best practices for scale development and validation. In addition, although the results may not be generalizable, the labor and vocational reasons assessed by the questionnaire are global topics, unlike academic reasons, which must consider the academic culture in each country. Ultimately, it is imperative to underscore that the examination and analysis of dental students’ motivations serve to tailor teaching strategies and educational methodologies within universities. Consequently, this contributes to the cultivation of a more dedicated, ethic and satisfied workforce, as suggested by Chate [30].

In conclusion, this research emphasizes the importance of labor, vocational, and academic reasons in students’ choice of dentistry. While the first two reasons are globally relevant, the Ecuadorian academic culture necessitates considering academic reasons. Future studies should explore variables like gender, university type, academic year, and the significance of academic motivations in choosing dentistry.

### Supplementary information

Supplementary Information

## Data Availability

The data that support the findings of this study are available from the corresponding author upon reasonable request.

## References

[1] Niven V, Cabot LB, Scambler S, Gallagher JE (2022). Dentistry as a professional career: the views of London’s secondary school pupils (2011-2017). Br Dent J.

[2] Nikolovska J, Eaton KA, Kenig N, Hysi D, Petricevic N (2020). Motivation to Follow a Career in Dentistry of Students in Three South-East European Countries. Acta Stomatol Croat.

[3] Du Toit J, Jain S, Montalli V, Govender U (2014). Dental students’ motivations for their career choice: an international investigative report. J Dent Educ.

[4] Khalaf ME, Abubakr NH, Alenezi H, Ziada H (2022). The motivation and confidence in choosing dentistry as a career amongst dental students: A mixed-methods study. Eur J Dent Educ J.

[5] Kaersgaard JLB, Christensen MK, Søndergaard PY, Naukkarinen J (2021). Gender differences in dentistry: A qualitative study on students’ intrinsic and extrinsic motivations for entering dentistry at higher education. Eur J Dent Educ J.

[6] Scepanovic T, Mati S, Ming ALC, Yeo P, Nguyen D, Aria M, et al. The global distribution of special needs dentistry across dental school curricula. Spec Care Dentist. 2024. 10.1111/scd.12973. 10.1111/scd.12973 38385902

[7] Gallagher J, Clarke W, Wilson N (2008). Understanding the motivation: a qualitative study of dental students’ choice of professional career. Eur J Dent Educ J.

[8] Katyal S, Kanji Z (2021). Students’ motivating influences for selecting dental hygiene and a 4-year degree: A retrospective study. Int J Dent Hyg.

[9] Bernabé E, Icaza JL, Delgado-Angulo EK (2006). Reasons for choosing dentistry as a career: a study involving male and female first-year students in Peru. Eur J Dent Educ J.

[10] Rashid H, Manoharan A, Abufanas S, Gallagher JE (2013). Motivation for a career in dentistry: the views of dental students in the United Arab Emirates. Int Dent J.

[11] Kanji Z. Dental hygiene baccalaureate degree education in Canada: Motivating influences and experiences. Can J Dent Hyg. 2010;44:147–155

[12] Al-Hallak KR, Nassani MZ, Heskul MM, Doumani MD, Darwish M (2018). Reasons for choosing dentistry as a career among dental students in Saudi Arabia. Eur J Dent.

[13] Karagir A, Khairnar MR, Adaki S, Dhole RI, Patil MC, Ingale A (2021). Assessment of the factors influencing dental students to choose dentistry as a career: A cross-sectional survey. Indian J Dent Res.

[14] Lukandu OM, Koskei LC, Dimba EO (2020). Motivations for a Career in Dentistry among Dental Students and Dental Interns in Kenya. Int J Dent.

[15] Rabeeah Z, Carreno JG, Kinney JS, Inglehart MR (2022). Career motivation and satisfaction of dental hygiene students in associate versus bachelor degree programs: A national survey. J Dent Educ.

[16] Yan X, Zhang X, Jinno Y, Tachibana K, Gao J, Koyano K (2014). Career choice and future design of dental students in China and Japan. Int Dent J.

[17] dos Santos BF, Nicolau B, Muller K, Bedos C, Zuanon ACC (2013). Brazilian dental students’ intentions and motivations towards their professional career. J Dent Educ.

[18] Kfouri MG, Moyses SJ, Moyses ST (2013). Women’s Motivation to Become Dentists in Brazil. J Dent Educ.

[19] Freire M, do CM, Jordao LMR, de Paula Ferreira N, de Fatima Nunes M, Queiroz MG (2011). Motivation Towards Career Choice of Brazilian Freshman Students in a Fifteen-Year Period. J Dent Educ.

[20] Aguiar CM, Pessoa MAV, Câmara AC, Perrier RA, de Figueiredo JAP (2009). Factors Involved in the Choice of Dentistry as an Occupation by Pernambuco Dental Students in Brazil. J Dent Educ.

[21] Folayan MO, Sofola OO, Khami MR, Esan AO, Popoola BO, Orenuga OO (2014). Study motives, career choices and interest in paediatric dentistry among final year dental students in Nigeria. BMC Med Educ.

[22] Vigild M, Schwarz E (2001). Characteristics and study motivation of Danish dental students in a longitudinal perspective. Eur J Dent Educ..

[23] Boateng GO, Neilands TB, Frongillo EA, Melgar-Quiñonez HR, Young SL (2018). Best Practices for Developing and Validating Scales for Health, Social, and Behavioral Research: A Primer. Front Public Health.

[24] Kishore K, Jaswal V, Kulkarni V, De D (2021). Practical Guidelines to Develop and Evaluate a Questionnaire. Indian Dermatol Online J.

[25] Gallagher JE, Clarke W, Eaton KA, Wilson NH (2007). Dentistry – a professional contained career in healthcare. A qualitative study of Vocational Dental Practitioners’ professional expectations. BMC Oral Health.

[26] Urbanaviciute I, Lazauskaite-Zabielske J, De Witte H (2021). Deconstructing Job Insecurity: Do its Qualitative and Quantitative Dimensions Add Up?. Occup Health Sci.

[27] Secretaría de Educación Superior, Ciencia, Tecnología e Innovación. Examen de Acceso a la Educación Superior. Boletín de Prensa No 6. 2021. https://www.educacionsuperior.gob.ec/examen-de-acceso-a-la-educacion-superior-primer-periodo-academico-del-ano-2021/.

[28] Watkins MW (2018). Exploratory Factor Analysis: A Guide to Best Practice. J Black Psychol.

[29] Brown TA (2015). Confirmatory Factor Analysis for Applied Research.

[30] Chate R (2019). Vocation and altruism vs business and profit. Am J Orthod Dentofac Orthop..

[31] Xu C, Gao L, Zhang S, Zhang J, Li C, Zhang D (2022). Motivations and future plans of the final year students in a Chinese dental school. BMC Med Educ.

[32] Kabil NS, Allam GG, Abd El-Geleel OM (2018). Motivational reasons for choosing dentistry as a professional career & factors affecting specialty choice among final year dental students. Future Dent J.

[33] Lone MA, Lone MM, Lone MA, Shaikh MS, Khan F, Soomro AH (2020). Motivational factors for pursuing dentistry as a profession in colleges of Karachi, Pakistan. J Pak Med Assoc.

[34] Herz MM, ElAyouti A (2022). Motives for studying dental medicine in Germany. Eur J Dent Educ J Assoc Dent Educ Eur.

[35] Niven V, Scambler S, Cabot LB, Gallagher JE (2023). Journey towards a dental career: the career decision-making journey and perceived obstacles to studying dentistry identified by London’s secondary school pupils and teachers. Br Dent J.

[36] Cheng FC, Wang LH, Wang YC, Chiang CP (2024). The influence of dentist parents on their children’s career decision-making for dentistry or medicine. J Dent Sci.

[37] Wolf TG, Otterbach EJ, Zeyer O, Wagner RF, Crnić T, Ilhan D (2021). Influence of Oral Health Care Systems on Future Career Environment of Dental Students in Europe. Int J Environ Res Public Health.

[38] Suhr D. Exploratory or Confirmatory Factor Analysis. 1st ed. Statistics and Data Analys. SAS Institute; 2006. 17 p.

[39] Rattray J, Jones MC (2007). Essential elements of questionnaire design and development. J Clin Nurs.

[40] Yusoff MSB, Arifin WN, Hadie SNH (2021). ABC of Questionnaire Development and Validation for Survey Research. Educ Med J..

[41] Scarbecz M, Ross JA (2002). Gender differences in first-year dental students’ motivation to attend dental school. J Dent Educ.

