# Supplementary material for: Unveiling motives for dentistry studies: psychometric validation of a comprehensive questionnaire among aspiring dental students
Source: BDJ Open. 2024 Mar 28;10:27. doi: 10.1038/s41405-024-00208-5 (PMC10979002; doi:10.1038/s41405-024-00208-5)
Supplement: Supplementary file 1 — Supplementary Information [file 41405_2024_208_MOESM1_ESM.pdf]

Table S1: Inter-item correlation matrix. Sample A: Model A

	Q10	Q11	Q12	Q13	Q14	Q1	Q2	Q3	Q4	Q21	Q23	Q24	Q5	Q7	Q8	Q15	Q18	Q20
Q10	**1,000**	**0,620**	**0,655**	**0,460**	**0,611**	0,180	0,324	0,246	0,285	0,030	-0,019	0,037	0,332	0,287	0,203	0,095	0,151	0,084
Q11	**0,620**	**1,000**	**0,640**	**0,494**	**0,537**	0,288	0,307	0,306	0,239	0,031	-0,012	-0,031	0,354	0,309	0,241	0,085	0,145	0,117
Q12	**0,655**	**0,640**	**1,000**	**0,574**	**0,589**	0,163	0,288	0,283	0,239	0,048	-0,056	-0,059	0,365	0,311	0,324	0,079	0,049	0,136
Q13	**0,460**	**0,494**	**0,574**	**1,000**	**0,545**	0,134	0,282	0,247	0,243	0,125	0,125	0,131	0,436	0,264	0,223	0,100	0,186	0,237
Q14	**0,611**	**0,537**	**0,589**	**0,545**	**1,000**	0,179	0,321	0,197	0,299	0,037	0,030	0,070	0,399	0,305	0,250	0,161	0,183	0,098
Q1	0,180	0,288	0,163	0,134	0,179	**1,000**	**0,522**	**0,537**	**0,496**	0,026	0,095	0,183	0,268	0,322	0,181	0,088	0,108	0,066
Q2	0,324	0,307	0,288	0,282	0,321	**0,522**	**1,000**	**0,619**	**0,587**	0,149	0,087	0,166	0,423	0,313	0,258	0,088	0,090	0,032
Q3	0,246	0,306	0,283	0,247	0,197	**0,537**	**0,619**	**1,000**	**0,629**	0,116	-0,013	0,185	0,357	0,359	0,254	0,074	0,130	0,094
Q4	0,285	0,239	0,239	0,243	0,299	**0,496**	**0,587**	**0,629**	**1,000**	0,098	0,024	0,126	0,537	0,435	0,294	0,078	0,056	0,052
Q21	0,030	0,031	0,048	0,125	0,037	0,026	0,149	0,116	0,098	**1,000**	**0,376**	**0,366**	0,023	-0,004	-0,044	0,172	0,107	0,279
Q23	-0,019	-0,012	-0,056	0,125	0,030	0,095	0,087	-0,013	0,024	**0,376**	**1,000**	**0,315**	0,030	0,022	0,021	0,108	0,030	0,197
Q24	0,037	-0,031	-0,059	0,131	0,070	0,183	0,166	0,185	0,126	**0,366**	**0,315**	**1,000**	0,045	0,000	0,021	0,092	0,109	0,119
Q5	0,332	0,354	0,365	0,436	0,399	0,268	0,423	0,357	0,537	0,023	0,030	0,045	**1,000**	**0,594**	**0,431**	0,060	0,030	0,058
Q7	0,287	0,309	0,311	0,264	0,305	0,322	0,313	0,359	0,435	-0,004	0,022	0,000	**0,594**	**1,000**	**0,460**	0,140	0,095	0,043
Q8	0,203	0,241	0,324	0,223	0,250	0,181	0,258	0,254	0,294	-0,044	0,021	0,021	**0,431**	**0,460**	**1,000**	0,090	0,110	0,048
Q15	0,095	0,085	0,079	0,100	0,161	0,088	0,088	0,074	0,078	0,172	0,108	0,092	0,060	0,140	0,090	**1,000**	**0,314**	**0,411**
Q18	0,151	0,145	0,049	0,186	0,183	0,108	0,090	0,130	0,056	0,107	0,030	0,109	0,030	0,095	0,110	**0,314**	**1,000**	**0,312**
Q20	0,084	0,117	0,136	0,237	0,098	0,066	0,032	0,094	0,052	0,279	0,197	0,119	0,058	0,043	0,048	**0,411**	**0,312**	**1,000**

Table S2: Inter-item correlation matrix. Sample B: Model B										
	Q1	Q2	Q3	Q4	Q10	Q11	Q12	Q14	Q21	Q23
Q1	**1,000**	**0,461**	**0,429**	**0,499**	0,151	0,140	0,155	0,155	0,142	0,140
Q2	**0,461**	**1,000**	**0,593**	**0,587**	0,221	0,210	0,218	0,144	0,143	0,130
Q3	**0,429**	**0,593**	**1,000**	**0,557**	0,187	0,180	0,209	0,097	0,151	0,198
Q4	**0,499**	**0,587**	**0,557**	**1,000**	0,276	0,216	0,262	0,166	0,154	0,123
Q10	0,151	0,221	0,187	0,276	**1,000**	**0,450**	**0,481**	**0,452**	0,081	0,071
Q11	0,140	0,210	0,180	0,216	**0,450**	**1,000**	**0,449**	**0,322**	0,065	0,050
Q12	0,155	0,218	0,209	0,262	**0,481**	**0,449**	**1,000**	**0,342**	0,072	0,066
Q14	0,155	0,144	0,097	0,166	**0,452**	**0,322**	**0,342**	**1,000**	0,143	0,110
Q21	0,142	0,143	0,151	0,154	0,081	0,065	0,072	0,143	**1,000**	**0,357**
Q23	0,140	0,130	0,198	0,123	0,071	0,050	0,066	0,110	**0,357**	**1,000**

Table S3: Inter-item correlation matrix. Sample B: Model C

	Q1	Q2	Q3	Q4	Q5	Q7	Q10	Q11	Q12	Q14	Q21	Q23
Q1	**1,000**	**0,461**	**0,429**	**0,499**	**0,327**	**0,306**	0,151	0,140	0,155	0,155	0,142	0,140
Q2	**0,461**	**1,000**	**0,593**	**0,587**	**0,418**	**0,315**	0,221	0,210	0,218	0,144	0,143	0,130
Q3	**0,429**	**0,593**	**1,000**	**0,557**	**0,362**	**0,257**	0,187	0,180	0,209	0,097	0,151	0,198
Q4	**0,499**	**0,587**	**0,557**	**1,000**	**0,452**	**0,396**	0,276	0,216	0,262	0,166	0,154	0,123
Q5	**0,327**	**0,418**	**0,362**	**0,452**	**1,000**	**0,509**	**0,351**	**0,261**	**0,318**	**0,248**	0,090	0,091
Q7	**0,306**	**0,315**	**0,257**	**0,396**	**0,509**	**1,000**	**0,332**	**0,297**	**0,343**	**0,258**	0,065	0,136
Q10	0,151	0,221	0,187	0,276	**0,351**	**0,332**	**1,000**	**0,450**	**0,481**	**0,452**	0,081	0,071
Q11	0,140	0,210	0,180	0,216	**0,261**	**0,297**	**0,450**	**1,000**	**0,449**	**0,322**	0,065	0,050
Q12	0,155	0,218	0,209	0,262	**0,318**	**0,343**	**0,481**	**0,449**	**1,000**	**0,342**	0,072	0,066
Q14	0,155	0,144	0,097	0,166	**0,248**	**0,258**	**0,452**	**0,322**	**0,342**	**1,000**	0,143	0,110
Q21	0,142	0,143	0,151	0,154	0,090	0,065	0,081	0,065	0,072	0,143	**1,000**	**0,357**
Q23	0,140	0,130	0,198	0,123	0,091	0,136	0,071	0,050	0,066	0,110	**0,357**	**1,000**
